# Phytochemical Analysis, Acute Toxicity, as well as Antihyperglycemic and Antidiabetic Activities of *Corchorus olitorius* L. Leaf Extracts

**DOI:** 10.1155/2022/1376817

**Published:** 2022-07-18

**Authors:** Rebecca Nakaziba, Aloysius Lubega, Jasper Ogwal-Okeng, Paul E. Alele

**Affiliations:** ^1^Department of Pharmacology and Therapeutics, Mbarara University of Science and Technology, P.O Box, Mbarara 1410, Uganda; ^2^Department of Pharmacology and Therapeutics, Lira University, P.O Box, Lira 1035, Uganda; ^3^Department of Pharmacology and Therapeutics, Makerere University College of Health Sciences, P.O Box, Kampala 7072, Uganda

## Abstract

*Backgroundand Aim*. Diabetes mellitus is a metabolic disorder that has no known cure with continuous endeavors to find a therapy for the condition. According to some studies, traditional leafy vegetables could prevent and manage diabetes by modifying the carbohydrate and lipid metabolism. In this study, a phytochemical analysis, acute toxicity, as well as antihyperglycemic and antidiabetic activity testing of the methanolic, diethyl ether, and aqueous leaf extracts of *Corchorus olitorius* L. was performed. *Materials and Methods*. Methanolic, diethyl ether, and aqueous leaf extracts of *Corchorus olitorius* L. were prepared by serial extraction. Phytochemical analysis was performed following standard methods. 52 mice were separated into 13 groups (A–M) of 4 and received extracts' doses ranging from 1000 mg/kg to 5000 mg/kg for the acute toxicity testing. For the antihyperglycemic and antidiabetic activities testing, 48 rats were divided into 8 groups of 6 and received 500 mg/kg of each extract. 10 mg/kg of glibenclamide and distilled water were used as controls. Data were analyzed using Prism GraphPad version 8.0.2 (263). *Results*. Phytochemical analysis revealed the presence of alkaloids, reducing sugars, saponins, and terpenoids. There were no acute toxicity signs observed in this study. *Corchorus olitorius* L. extracts demonstrated moderate antihyperglycemic and antidiabetic activities. The methanolic extract exhibited the highest degree of antihyperglycemic activity. However, there was no statistically significant difference between the extracts and the negative control (*p* > 0.05), but with glibenclamide (*p* < 0.01). *Conclusion*. *Corchorus olitorius* L. is a safe and potential postprandial antidiabetic vegetable that could minimize the rise in blood glucose after a meal. We therefore recommend further investigations into the antidiabetic properties of the vegetable using purified extracts.

## 1. Introduction

*Corchorus olitorius* L. (*C. olitorius*), also known as Tossa jute in English, is a traditional medicinal vegetable belonging to the genus *Corchorus* and the family of Tiliceae [1]. The vegetable is prevalent in northern Australia, tropical Africa, and India [1]. In these places, *C. olitorius* leaves commonly are served as a vegetable frequently in stews eaten with staple starchy foods [1]. *C. olitorius* is an annual herb growing to a height of 2.4 m [1]. It has alternating, ﬁnely indented margin leaves. *C. olitorius* has small yellow ﬂowers comprising of ﬁve petals that form a brown multiseeded pod later in life [1]. It is proliferated by seeds [2] much as it can be accepted as a wild vegetable in crop fields or cultivated in family gardens [2]. *C. olitorius* flourishes best throughout rainy seasons, nonetheless it is drought resilient [1, 2]. In West Africa, *C. olitorius* is used to treat malaria, typhoid fever, female infertility, heart failure, and ulcers [1, 3, 4]. In addition, it is used as a therapy for fevers, colds, constipation, and tumors [5]. The vegetable is also reported to have antiobesity [6, 7], anti-inflammatory [8, 9], antimicrobial [10, 11], and gastroprotective properties [12] along with antidiabetic effects [7, 13, 14].

Diabetes mellitus (DM), a disorder of mainly glucose metabolism, is one of the most common noncommunicable diseases (NCDs). In 2015, DM caused 5 million deaths and an estimated 12% of global expenditure [15–17]. The disease occurs in two types, that is, 1 and 2. Type 1 accounts for 2–10% of all diabetic cases in the world [18] and for >85% of diabetes registered amongst youth below 20 years of age [19]. It occurs as a result of autoimmune destruction of pancreatic beta cells with a subsequent total insulin deficiency [18]. Type 2 results from insensitivity to insulin and/or inadequate compensatory insulin secretion with over 90% of all diabetic cases in the world [15]. In Africa, diabetes occurred among 7.1% of the population in 2014, and the overall cost of DM was USD 19.45 million in 2015 [20]. More than 90% of diabetic cases in Africa are type 2 [20, 21]. In Uganda, the prevalence of diabetes was 1.9% in 2016 [22]. Diabetes is managed by dietary modification, exercise, and pharmacotherapy [23]. However, there is as yet no known cure for DM. This has driven the affected individuals' world over to opt for alternative medication including traditional herbal remedies, while a lot of research is being conducted to evaluate and document plants with glycemic effects [24–31]. In Uganda and other parts of the world, studies have revealed a number of plants that are used by communities in the management of diabetes [32, 33]. According to a study conducted by Momo et al [31], traditional leafy vegetables could prevent and manage diabetes by modifying the carbohydrate and lipid metabolism. *C. olitorius* has indicated a reduction in serum blood glucose levels in a few studies [34].

In the current study, we performed a phytochemical analysis, acute toxicity, as well as antihyperglycemic and antidiabetic activity testing of the methanolic, aqueous, and diethyl ether extracts of *C. olitorius* leaves in laboratory animals. This was done to confirm some of the reports about the vegetable as well as to assess its safety.

## 2. Materials and Methods

### 2.1. Laboratory Animals

Laboratory mice and rats from the animal house of the Department of Pharmacology and Therapeutics, Makerere College of Heath Sciences, were used for this study. The animals (male and female) were housed in wooden cages at temperature between 25 and 28°C with a normal 12-hour/12-hour light/dark cycle for at least 2 weeks before the study. They were fed with standard rat feeds and water. Their weight ranged from 18 to 31 g and 128 g to 221 g for mice and rats, respectively. Pregnant and breastfeeding animals were excluded from the study.

### 2.2. Extract Preparation

The *C. olitorius* leaves were harvested from Olago village, Lira district (northern Uganda), and packaged in polyethene bags. They were then transported to the Department of Pharmacology and Therapeutics, Makerere University College of Health Sciences, where they were washed clean and spread to air-dry inside the laboratory to a constant weight (900 g). The fully dried leaves were then ground to a course powder and extracted by means of solvents (diethyl ether, methanol, and distilled water) in a serial extraction method as described by Das, Tiwari, and Shrivastava [35]. This was done so as to extract compounds of varied polarity. Briefly, 600 g of *C. olitorius* leaves powder were soaked in 3 L of diethyl ether (BDH AnalaR) in a flat bottomed conical flask and tightly closed while shaking every 6 hours for two days (48 hours.). The resultant solution was filtered using Whatman filter paper (No. 1), and the filtrate was concentrated by means of a rotary evaporator at 20–30°C. The remains were then spread in the laboratory and allowed to dry. After drying, it was soaked in 3 L of methanol (UltraPure Solutions, Inc. 11485, Commercial Parkway, Castroville, CA 95012), and the process was repeated as with diethyl ether. Last, the dry residue was soaked in 3 L of distilled water while shaking every 6 hours for two days (48 hours). The resultant solution was also filtered using Whatman filter paper (No. 1) and freeze dried. After evaporation of the solvents, the extracts were accordingly labeled and stored in a refrigerator at −80°C. A voucher specimen was kept at a herbarium at the Department of Botany, Makerere University, Kampala, Uganda (# 50906).

### 2.3. Phytochemical Analysis

The phytochemical analysis was performed as follows.

#### 2.3.1. Tannins

2 mL of 5% FeCl_3_ were added to 2 mL of aqueous extract and observed for a yellow/brown precipitate formation [36].

#### 2.3.2. Alkaloids

1.5 mL of 1% HCl was added to 2 mL of the methanol extract filtrate, the solution was heated in a waterbath (5 minutes), and then 6 drops of Mayor's reagent was added and observed for an orange precipitate formation [37].

#### 2.3.3. Saponins

2 g powder of the aqueous extract was mixed with few drops of olive oil, shaken vigorously, and observed for formation of a stable persistent froth and an emulsion [38].

#### 2.3.4. Cardiac Glycosides

To 2 mL methanol extract filtrate, 1 mL glacial acetic acid and 1-2 drops of FeCl_3_ were added followed by 1 mL of concentrated H_2_SO_4_. Formation of a brown ring at the interface indicated the presence of deoxy-sugar [39, 40].

#### 2.3.5. Terpenes

To 2 mL of the aqueous extract, 5 mL CHCl_3_, 2 mL acetic anhydride, and a drop of concentrated H_2_SO_4_ were added carefully to form a layer. Formation of a reddish/brown coloration at the interface indicated the presence of terpenes [41].

#### 2.3.6. Reducing Sugars

To 2.5 mL of Benedict's solution was added 0.5 g of the aqueous extract in a test tube. The mixture was warmed over a waterbath for about 5 minutes. Observation of green/red or yellow coloration indicated the presence of reducing sugars [42].

#### 2.3.7. Nonreducing Sugars

3 mL of the aqueous extract was mixed with 2 drops of dilute iodine solution and boiled for 5 minutes. Observation of blue color that disappeared upon boiling and reappeared upon cooling indicated the presence of nonreducing sugar [43].

### 2.4. Acute Toxicity Testing

The extracts were orally administered following the protocol laid down by the Organization for Economic Cooperation and Development [44], where the dose of the extract per animal body weight was calculated and administered to the animals suspended in distilled water. The animals were observed for mortality, behavioral changes, physical appearance, injury, and pain after 30 minutes, 1 hour, 2 hours, 4 hours, 8 hours, and once daily for 13 days. The control group was only given distilled water. Observations were recorded per group of animals. The experiment was performed as follows: a total of 52 mice (average weight 25 g) were divided into thirteen (13) groups (A–M) of four (4). Groups A–D, E–H, and I–L received 1000 mg/kg, 2000 mg/kg, 4000 mg/kg, and 5000 mg/kg of the diethyl ether, methanol, and aqueous extracts, respectively. Last, group M received 1 mL of distilled water.

### 2.5. The Antihyperglycemic and Antidiabetic Tests

This was performed as described by Mushtaq et al. [45]with minor modifications as follows. The rats were fasted overnight and randomly selected and distributed into eight groups (*n* = 6). To one of the groups, 1 mL of distilled water was orally administered. To three of the groups (for the antihyperglycemic test), the methanolic, diethyl ether, and aqueous extracts of *C. olitorius* (500 mg/kg body weight for each extract) were orally administered. After one hour, the animals were fed with glucose (2 g/kg body weight) [45]. To another set of three groups (for the antidiabetic test), alloxan (60 mg/kg; i.v) was given prior (3 days) to the study in order to induce diabetes. These too were given in 500 mg/kg of body weight of each of the extracts. The last group received glibenclamide (10 mg/kg body weight). A blood sample was then collected by pricking the tail vein of the rat using a scalp vein needle. Blood was gently milked, dropped on a glucose strip, and read using a glucometer (Code-free blood glucose test strips, Biosenser, Inc, Korea and Code free, Yeongtong-dong Yeontong-du, Suwoni-si, Kyonggi-go, Korea). Blood was withdrawn from the tail vein at 0, 30, 60, 90, 120, and 150 minutes of glucose administration for the antihyperglycemic test and the same time interval following test drug administration for the antidiabetic test. The glucose level is recorded in a table as indicated by the glucometer.

### 2.6. Data Management

Data was analyzed by Prism GraphPad version 8.0.2 (263) and expressed as mean + standard deviation. Significance level was set at *p* ≤ 0.05 at 95% confidence interval (CI). The results are presented as tables and figures.

## 3. Results

### 3.1. Phytochemical Analysis

Phytochemical analysis revealed the presence of alkaloids, reducing sugars, and saponins among others (Table 1).

### 3.2. Acute Toxicity Testing

There were no observed acute toxicity effects of the *C. olitorius* extracts at the doses tested. Neither behavioral or appearance changes nor mortality was observed in all the groups. All the animals appeared and behaved normally (Tables 2 and 3).

### 3.3. Antihyperglycemic and Antidiabetic Activities Testing

**Figure 1:**
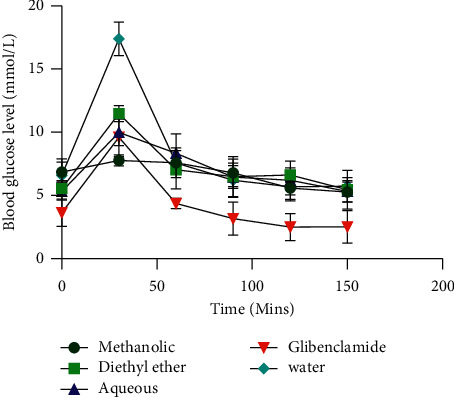
Blood glucose levels for the antihyperglycemic test.

To assess the antihyperglycemic activity of *C. olitorius,* the methanolic, diethyl ether, and aqueous extracts of *C. olitorius* leaves along with distilled water and glibenclamide were administered to rats an hour before glucose administration. The blood glucose level was then taken at different time intervals using a glucometer. For the antidiabetic test, the test animals were given alloxan three days before the experiment. *C. olitorius* extracts moderately exhibited antihyperglycemic and antidiabetic effects, although not comparable to glibenclamide (Table 4 and Figure 1).

*P* value <0.05 was compared with positive control but >0.05 with negative control for the antihyperglycemic test; *P* value = <0.0001 was compared with water in the antidiabetic test (unpaired *t*-test with Welch's correction) for all the extracts. The overall *P* value calculation using the one-way ANOVA and multiple *t*-test was below 0.05 showing a statistically significant difference between the *C. olitorius* extracts and glibenclamide, whereas it was above 0.05 in comparison with water indicating no significant difference. However, the multiple *t*-test per time interval revealed a statistically significant difference between *C. olitorius* extracts and water at 30 minutes of glucose administration (*P* value <0.05).

## 4. Discussion

Phytochemical analysis revealed the presence of alkaloids, glycosides, saponins, tannins, terpenoids, and reducing sugars (Table 1). This finding was consistent with a related study in India [46] and Bangladesh [47] but not in West Africa [48]. In another related study, alkaloids, tannins, and resins were found [34]. The discrepancy in phytochemical composition could be attributed to the differences in the climate of the regions. In addition, there was no observed acute toxicity effect of *C. olitorius* extracts seen in the current study (Table 3). This finding is similar to a set of related studies in Bangladesh and Nigeria where there were no observed acute toxicity effects in doses comparable to those of this study [47, 49]. In another related study, *C. olitorius* did not show any toxicity using enzyme markers in alloxan-induced diabetic rats [34]. This finding also supports the results of the present study. This implies that the vegetable is safe for consumption. To assess the antihyperglycemic activity of *C. olitorius* leaf extracts, the controls and the *C. olitorius* extracts were administered an hour before glucose ingestion. Glibenclamide registered the lowest blood glucose at 0 hour of glucose administration. This was partly in agreement with the findings of Maxwell et al. in Nigeria [14] who tested the antidiabetic potential of the ethanolic seed extract of *C. olitorius* and found glibenclamide to have a higher blood glucose lowering potential than the *C. olitorius* extract in normal glycemic rats. Meanwhile, there was no significant difference between the blood glucose levels of the *C. olitorius* extracts in comparison with the negative control at 0 hour. However, the blood glucose level for the methanolic extract was slightly higher than that of the negative control at 0 hour (Table 4). This indicates that the extracts have a minimal effect on baseline blood glucose. In an oral glucose tolerance test (OGTT) performed by Maxwell et al. [14], the ethanolic *C. olitorius* seed extract demonstrated a significant decrease in the blood glucose level from 30 minutes to 2 hours (*P* < 0.001). Similarly, in the current study, the different *C. olitorius* extracts demonstrated significant antihyperglycemic activity at 30 minutes of glucose ingestion (*p* < 0.01). Nevertheless, the overall *p* value (*p* > 0.05) in comparison with the negative control for the entire test was not statistically significant. This could be attributed to the fact that they (Maxwell et al.) [14] used the *C. olitorius* seed extract, yet the current study used leaves. There is also a probability that *C. olitorius* seeds exhibit a higher antihyperglycemic potential than the leaves. Comparison between the *C. olitorius* extracts and glibenclamide revealed that glibenclamide had a higher antihyperglycemic activity (*p* < 0.01) except with the aqueous extract at 30 minutes (*p*=0.63). This too was similar to the findings of Maxwell et al. [14]. Their glibenclamide group had the highest reduction in blood glucose level among the test animals. This could be justified by the fact that glibenclamide is an already purified compound unlike the extracts. In another study conducted by Arise et al. [13], to establish the antidiabetic properties of *C. olitorius* in alloxan-induced diabetic rats, the ethanolic leaf extract exhibited antidiabetic activity. This finding relates to the outcome of the current study, although a methanolic extract was used in the current study. In addition, according to a study that tested the hypoglycemic effect of *C. olitorius* in albino rats, the methanolic extract demonstrated hypoglycemic activity, thereby, supporting the findings of the current study [7]. In a related study conducted by Sanjida et al. in Bangladesh [47], *C. olitorius* showed a dose-dependent reduction in the blood glucose level in an oral glucose tolerance test (OGTT) among test animals. This as well is in agreement with the first one hour findings of the current study. Furthermore, a study was conducted by Oboh et al. [50] to identify the antidiabetic potential of *C. olitorius*, and the plant demonstrated antidiabetic activity in type 2 diabetic rats. This outcome also is in agreement with the findings of the current study, although the current effect was minimal. All the extracts in the present study moderated the rise in plasma glucose an hour after oral ingestion of glucose by the test animals. The methanolic extract showed the greatest antihyperglycemic activity within the first one hour. However, after the first one hour, there was no significant difference between the plasma glucose level of the extracts and water signifying a short-lived antihyperglycemic activity of the extracts. They therefore cannot be considered for long-term control of high blood sugar. In addition, the antihyperglycemic and antidiabetic activities of the extracts were less when compared to those of glibenclamide. This is attributable to the fact that glibenclamide is a pure compound. The antihyperglycemic and antidiabetic activities of *C. olitorius* are ascribed to the presence of alkaloids, which are believed to induce glucose uptake by pancreatic beta cells [51].

## 5. Conclusion and Recommendation

*C. olitorius* L. has moderate antihyperglycemic and antidiabetic activities with no toxic effects. Further in-depth studies to investigate the antihyperglycemic, antidiabetic, pharmacodynamics, and the pharmacokinetic properties of *C. olitorius* L. leaf extracts as a potential nutraceutical amongst diabetic patients should be performed using purified extracts.

## Figures and Tables

**Table 1:** Phytochemical analysis of *C. olitorius*.

Phytochemicals	Present (+)/absent (−)
Alkaloid	+
Glycoside	+
Reducing sugar	+
Nonreducing sugar	−
Saponins	+
Tannins	+
Terpenoids	+

**Table 2:** Extract doses given.

Group	Extract	Dose (mg/kg)
A	Methanol	1000
B	Methanol	2000
C	Methanol	4000
D	Methanol	5000
E	Diethyl ether	1000
F	Diethyl ether	2000
G	Diethyl ether	4000
H	Diethyl ether	5000
I	Aqueous	1000
J	Aqueous	2000
K	Aqueous	4000
L	Aqueous	5000
M	Distilled water	0

**Table 3:** Acute toxicity of *C. olitorius* extracts.

Groups	Dose	Mortality (#)	Behavioral change (#)	Change in physical appearance (#)	Expression of pain (#)
A, E, I	1000 mg/kg	0	0	0	0
B, F, J	2000 mg/kg	0	0	0	0
C, G, K	4000 mg/kg	0	0	0	0
D, H, L	5000 mg/kg	0	0	0	0
M	0 mg/kg	0	0	0	0

^#^Number.

**Table 4:** The mean values of the plasma glucose level per group with the standard deviations.

T	M1	DE1	A1	M2	DE2	A2	Gl	W
0	6.850 ± 0.797	5.967 ± 0.625	5.317 ± 0.688	6.160 ± 0.953	5.900 ± 0.490	5.920 ± 0.746	3.633 ± 1.098	6.538 ± 1.327
30	7.767 ± 0.441	11.467 ± 0.644	9.983 ± 1.057	7.600 ± 0.797	5.460 ± 0.783	6.820 ± 1.062	9.600 ± 1.534	17.383 ± 1.326
60	7.583 ± 1.186	7.033 ± 1.529	8.333 ± 1.537	7.360 ± 1.555	5.860 ± 1.106	5.560 ± 0.757	4.367 ± 0.413	7.567 ± 0.378
90	6.783 ± 1.091	6.483 ± 1.592	6.450 ± 0.907	6.940 ± 1.001	6.620 ± 0.746	7.720 ± 1.893	3.167 ± 1.303	6.217 ± 1.353
120	5.583 ± 0.542	6.617 ± 0.571	6.217 ± 1.530	7.220 ± 0.576	6.680 ± 0.847	7.760 ± 0.358	2.500 ± 1.081	5.683 ± 1.118
150	5.267 ± 0.799	5.467 ± 1.549	5.317 ± 0.854	6.440 ± 0.777	7.400 ± 0.812	6.440 ± 0.643	2.517 ± 1.300	5.733 ± 0.668

T, time in minutes; M1, DE1, and Aq1, methanol, diethyl ether, and aqueous for extracts antihyperglycemic test; M2, DE2, and Aq2, methanol, diethyl ether, and aqueous extracts for the antidiabetic test; Gl, glibenclamide; W, distilled water.

## Data Availability

The data used to support this study are available from the corresponding author upon request.
